# Abnormal PITX1 gene methylation in adolescent idiopathic scoliosis: a pilot study

**DOI:** 10.1186/s12891-018-2054-2

**Published:** 2018-05-09

**Authors:** Benlong Shi, Liang Xu, Saihu Mao, Leilei Xu, Zhen Liu, Xu Sun, Zezhang Zhu, Yong Qiu

**Affiliations:** Spine Surgery, the Affiliated Drum Tower Hospital of Nanjing University Medical School, Zhongshan Road No. 321, Nanjing, 210008 China

**Keywords:** Adolescent idiopathic scoliosis, Pituitary homeobox 1, Gene methylation, Pyrosequencing

## Abstract

**Background:**

The gene of pituitary homeobox 1 (PITX1) has been reported to be down-regulated in adolescent idiopathic scoliosis (AIS), of which the cause has not been well addressed. The abnormal DNA methylation was recently assumed to be an important mechanism for the down-regulated genes expression. However, the association between PITX1 promoter methylation and the etiology of AIS was not clear.

**Methods:**

The peripheral blood samples of 50 AIS patients and 50 healthy controls were collected and the genomic DNA was extracted. The pyrosequencing assay was used to assess the methylation status of PITX1 promoter and real-time quantitative polymerase chain reaction (PCR) was used to detect the PITX1 gene expression. Comparison analysis was performed using independent t test and Chi-square tests, while correlation analysis were performed with 2-tailed Pearson coefficients.

**Results:**

The mean methylation level was (3.52 ± 0.96)% in AIS and (1.40 ± 0.81)% in healthy controls (*P* < 0.0001). The PITX1 gene expression was 0.15 ± 0.08 in AIS and 0.80 ± 0.55 in healthy controls (*P* < 0.0001). The comparative analysis showed significant difference in age (*P* = 0.021) and Cobb angle of the main curve (*P* = 0.0001) between AIS groups with positive and negative methylation. The methylation level of 6 CpG sites in PITX1 promoters was significantly associated with Cobb angle of the main curve (*P* < 0.001) in AIS. No statistical relationship between PITX1 promoter methylation and gene expression was found in AIS (*P* = 0.842).

**Conclusion:**

Significantly higher methylation level and lower PITX1 gene expression are found in AIS patients. PITX1 methylation is associated with Cobb angles of the main curves in AIS. DNA methylation thus plays an important role in the etiology and curve progression in AIS.

## Background

Adolescent idiopathic scoliosis (AIS) is a three-dimensional spinal deformity commonly seen in females with prevalence ranging from 1 to 3% in adolescents [1, 2]. The origin and cause of AIS remain obscure although there are several proposed etiological hypotheses such as genetic variations [2, 3], abnormal bone mineral density [4, 5], asymmetrical growth and biomechanical conditions [6, 7], abnormal changes of the nervous system [8, 9], and hormonal variations [10, 11]. In addition, Fendri et al. [12, 13] comparatively analyzed the gene expression patterns of AIS osteoblasts and healthy osteoblasts by microarray analysis, in which the gene of pituitary homeobox 1 (PITX1) was reported to be down-regulated more than 1.5-fold in AIS. This is an interesting observation of which may potentially shed lights on the etiopathogenesis of AIS.

Even though the significantly lower expression of PITX1 gene in AIS has been previously reported [12], the cause of this abnormality remains unclear. Recently, the DNA methylation has been proposed as an important epigenetic mechanism operating at the interface between genome and environment to regulate phenotypic plasticity with complex regulations across the genome during the first decade of life [14]. DNA methylation may be the most suitable epigenetic mark for large-scale epidemiological studies since methyl groups are covalently bounded to CpG dinucleotides and are not lost during routine DNA extraction. This opens the possibility of exploiting existing DNA biomarkers in order to discover epigenetic risk factors for complex disease [15]. It was hypothesized that the abnormal DNA methylation might play an important role in the down-regulation of PITX1 gene expression, serving as an epigenetic factor leading to the occurrence and development of AIS.

The purposes of the current study were: 1) to evaluate the DNA methylation of PITX1 promoter in AIS; 2) to investigate the relationship between abnormal DNA methylation and PITX1 gene expression; and 3) further to analyze the role of abnormal DNA methylation in the occurrence and development of AIS through genetic and epigenetic perspectives.

## Methods

### Materials

TIANamp Blood DNA kit (Tiangen),DNA isolation kit (Qiagen AB, Solna, Sweden), DNA Methylation Kit™ (Zymo Research, HiSS Diagnostics, CA, USA), ZymoTaq™ Pre mix (Zymo Research, HiSS Diagnostics, CA, USA), PyroMark® Q24 pyrosequencer (Qiagen, Germany), RNeasy Plus Mini kit (QIAGEN), ReverTra Ace qPCR RT kit (Toyobo), SYBR-Green PCR master mix (Toyobo).

### Subjects

New AIS patients seen in our scoliosis center from January 2014 to January 2015 were included in the study. The inclusion criteria were: 1) patients with standing whole spine x-rays showing single thoracic or thoracolumbar curve (Cobb angle ranging from 10 deg. to 50 deg.); 2) age between 10 to 17 years; 3) with MRI of whole spine showing no additional abnormality of spinal cord or vertebrae; and 4) with complete demographic data including sex, age, etc. The exclusion criteria were as follows: 1) patients with previous spinal surgery; and 2) with any signs of growth abnormalities, neurological abnormality, skeletal dysplasia, dwarfism, or recognized genetic syndrome, for example Marfan syndrome. Finally, a total of 50 AIS patients were included, and 50 sex matched and age matched healthy controls were recruited in the study. Considering the association between CpGs methylation of PITX1 gene and cancer [16, 17], the possibility of cancer in both AIS and control groups were excluded with physical examination and inquiry of family history. The peripheral blood samples were collected from all the subjects. This retrospective study was approved by the ethics review board of the affiliated Drum Tower Hospital of Nanjing University, and the methods were carried out in accordance with the approved guidelines. All participating subjects or their legal guardians signed the written informed consents.

### Methods

#### DNA extraction and sodium bisulfite modification

Genomic DNA was extracted from blood samples using TIANamp Blood DNA kit (Tiangen). Bisulfite modification was done with 500 ng samples of DNA using DNA Methylation Kit™ (Zymo Research, HiSS Diagnostics, CA, USA). The samples were then eluted in 50 μL of elution buffer and stored at − 20°C.

#### Generation of PCR products and pyrosequencing

**Fig. 1:**
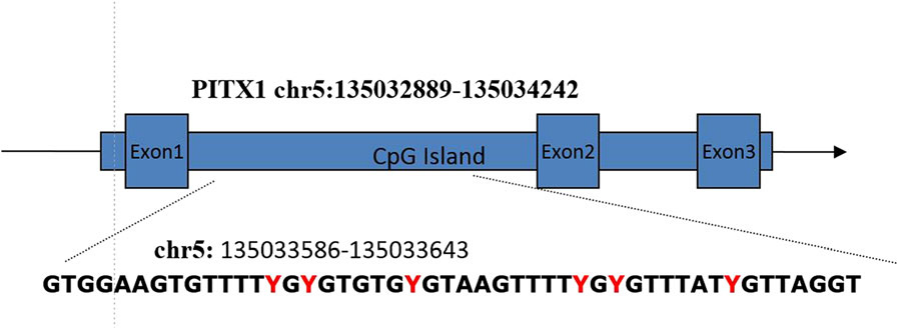
Schematic methylation sites of PITX1 gene promoter area

**Fig. 2.**
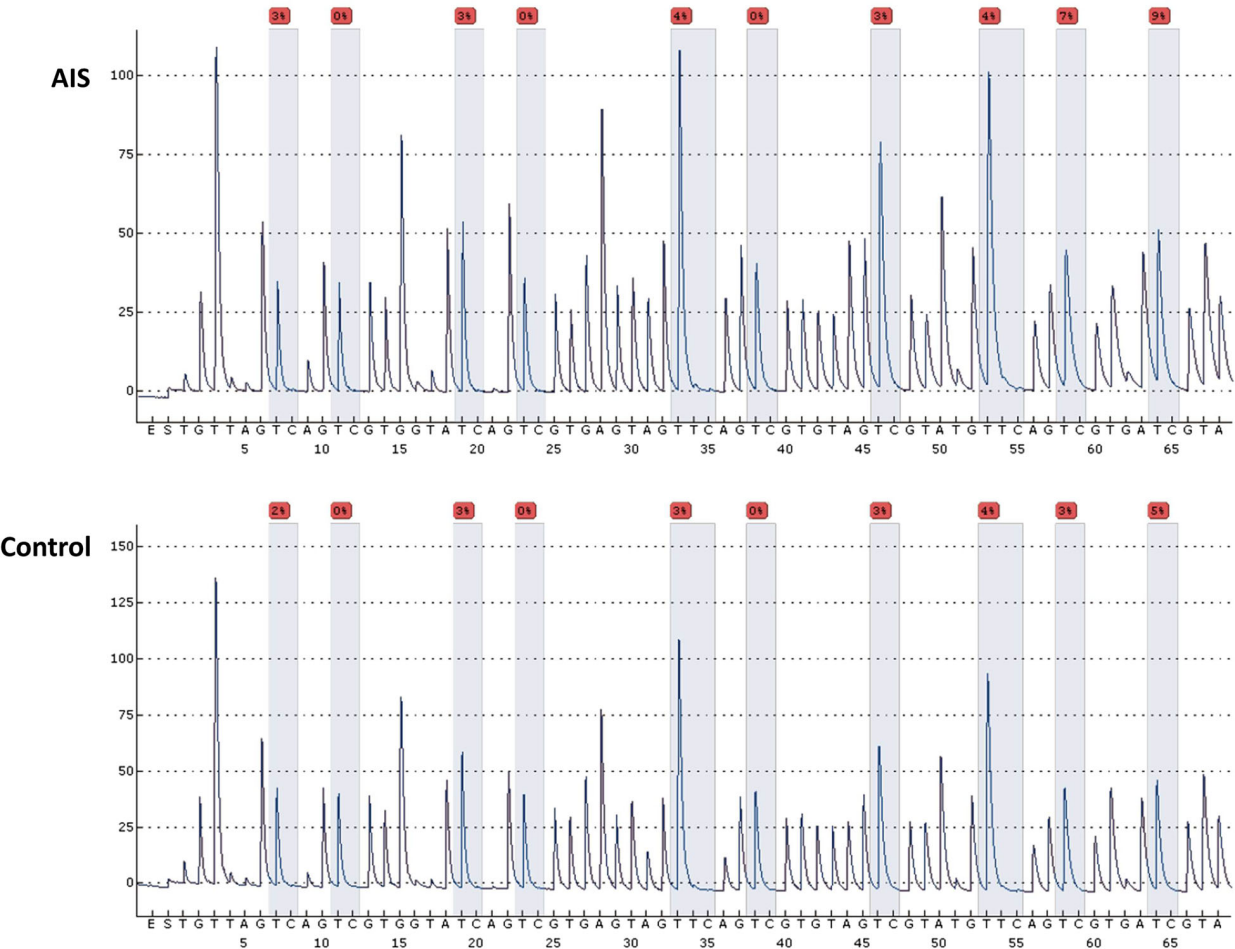
Demo cases of methylation sites between Exon 1 and Exon 2 detected by pyrosequencing after bisulfite conversion. The AIS patients showed higher methylation level than controls

Pyrosequencing [18] was a sequence-by-synthesis technique based on the fact that DNA polymerization could be monitored by measuring pyrophosphate production, which was detected by light. The pyrosequencing assay was used to assess the methylation status of PITX1 promoter (chr5:135032889–135,034,242) (Fig. 1). This assay detected the level of methylation in the region Chr5:135033586–135,033,643 of the PITX1 gene (chr5:134,363,424–134,369,964). The polymerase chain reaction (PCR) and pyrosequencing primers were designed using PyroMark Assay Design 2.0. The PCR volume was 20 μL, and was incorporated with 0.5 μM forward and reverse primers respectively, 10 μl ZymoTaqTM Pre mix (Zymo Research, HiSS Diagnostics, CA, USA) and 2 μl bisulfite-modified DNA. The forward and reverse primer sequences were 5′- GAT TAA GAG GGG TTG TTA GTT TAA TT -3′ and 5′-biotin- CCC CAA AAA CCT CAA AAA CTT TCT TTT TC -3′, respectively. PCR testing was carried out at 95°C for 10 min, followed by 40 cycles of 95°C for 30s, 55°C for 30s, and 72°C for 1 min, and a final extension at 72°C for 10 min. PCR product quality was confirmed by electrophoresis with 1% agarose gels with ethidium bromide staining. The pyrosequencing primer was 5′- AGT TGT AGG TTA GTG TAT TTG TTA -3′. Pyrosequencing was subsequently carried out in the PyroMark® Q24 pyrosequencer (Qiagen, Germany) (Fig. 2). The degree of methylation of all 6 CpG sites were automatically analyzed by the PyroMark® Q24 software. The exact frequency of methylation could be determined by the resulting peak patterns, which differed between methylated and unmethylated samples [19].

### RT-PCR

Total RNA was extracted from blood samples using RNeasy Plus Mini kit (QIAGEN). A total of 1 μg RNA was reversely transcribed using ReverTra Ace qPCR RT kit (Toyobo) according to the manufacturer’s guideline. The resulting cDNA samples were amplified by real-time PCR using primers specific for PITX1 gene expression and GAPDH served as an internal control. Primers were as follows: PITX1: forward, 5′-GTG GCG TAA GCG CGA GCG TAA-3′; Reverse, 5′- GAC AGC GGG CTC ATG GAG TTG AAG-3′. GAPDH (housekeeping gene): forward, 5’-CGG ATT TGG TCG TAT TGG G-3′ and reverse, 5’-CTG GAA GAT GGT GAT GGG ATT-3′. SYBR-Green PCR master mix (Toyobo) containing 333 nM of each forward and reverse primer and 1 μl cDNA were transferred into a Eco plate (Illumina). PCR was performed on Eco™ real-time PCR system (Illumina). The amplification program was as follows: 95°C for 1 min, followed by 40 cycles of denaturation at 95°C for 15 s and annealing at 60°C for 30s. Melting curve was used to monitor specificity of PCR products, which was further quantitated by 2^-△△Ct^ methods.

### Statistical analysis

Clinical and biological variables were compared using independent t test and Chi-square tests. Correlation analysis were performed using the 2-tailed Pearson coefficients of correlation. Statistical significance was set at a level of *p* < 0.05. All statistical analysis was performed with SPSS Statistics 17.0 software (SPSS, Inc., Somers, NY, USA).

## Results

### Comparison between healthy controls and AIS

**Fig. 3:**
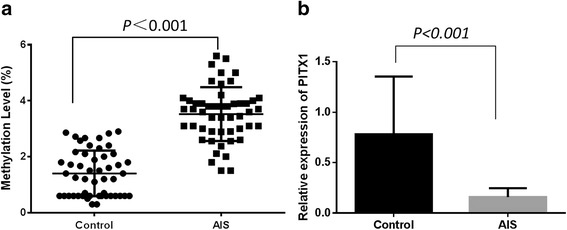
Comparison analysis of methylation level and gene expression between control and AIS groups

A total of 50 AIS patients and 50 healthy controls were included in the study. Overall, the mean methylation level was (3.52 ± 0.96)% in AIS patients and (1.40 ± 0.81)% in healthy controls (*P* < 0.0001) (Fig. 3a). Besides, the PITX1 gene expression was 0.80 ± 0.55 in healthy controls and 0.15 ± 0.08 in AIS patients, respectively (*P* < 0.0001) (Fig. 3b).

### Comparison between positive and negative methylation AIS groups

Two subgroups were defined for statistical analysis according to the average of CpG methylation status: the negative methylation group (defined as methylation level < 3.8%) and the positive methylation group (defined as methylation level ≥ 3.8%). A total of 22 AIS patients were divided into positive methylation group while 28 patients were in negative methylation group, respectively (Table 1). The comparative analysis between 2 groups showed no significant difference in sex (*P* = 0.308). However, significant difference was found in age (*P* = 0.021) and Cobb angle of main curve (*P* = 0.0001) between groups. The PITX1 gene expression was 0.07 ± 0.04 and 0.24 ± 0.11 in positive and negative methylation groups, revealing significant difference between 2 groups (*P* < 0.0001).

**Table 1:** Comparison analysis of the clinical characteristics between AIS patients with positive and negative methylation of PITX1 promoter

Parameters		Methylation		*P* value	χ2 value
		Positive	Negative		
Age (y)	10–13	14	8	0.021^*^	6.148
	14–16	8	20		
Sex	Female	19	27	0.308	1.696
	Male	3	1		
Cobb angle of main curve (°)	≤30	2	18	0.0001^*^	15.639
	>30	20	10		

^*^P < 0.05

### Correlation between methylation status and clinical characteristics in AIS

The methylation level of the 6 CpG sites in PITX1 promoters was significantly associated with Cobb angle of main curve (*P* < 0.001) in AIS patients. No significant correlation was found between methylation status and other clinical parameters including age, height or weight (*P* > 0.05 for all). Notably, no statistical linear relationship between PITX1 promoter methylation and gene expression was found (*P* = 0.842).

## Discussion

Several etiological hypotheses including both genetic and environmental factors have recently been proposed for the occurrence of AIS [12, 14]. Being reversible and heritable modifications, epigenetic markers such as DNA methylation are important to reflect the interactions between genetic factors and environmental exposures [20, 21]. We further assumed that the DNA methylation can also be up- or down-regulated in AIS patients, which serves as an important mechanism of this longitudinal and complex spinal deformity. Therefore, this study compared the DNA methylation between AIS patients and normal controls, aiming to explore the etiology of AIS through genetic and epigenetic aspects.

According to previous studies, the PITX1 is a member of the RIEG/PITX homeobox transcription factors and acts as a transcriptional regulator involved in basal and hormone-regulated activity of prolactin [22]. Members of this family are involved in organ development and left-right asymmetry [23]. The PITX1 abnormality is associated with many bone related diseases including congenital clubfoot, with or without deficiency of long bones and/or mirror-image polydactyly, and Liebenberg syndrome [24–26]. Besides, the abnormal PITX1 gene expression was also found in other pathological conditions such as lung cancer, cutaneous malignant melanoma and others [27, 28]. Hence, there is potential association between PITX abnormality and AIS. Recently, Fendri et al. [12] identified several genes involved in various bone regulatory and developmental pathways and showed many of them can be grouped into clusters to participate in a particular biological pathway [12]. Fendri’s study proved that the PITX1 gene expression was significantly lower in AIS patients. However, the cause of reduced expression of PITX1 and its corresponding mechanism in AIS remained unknown.

The current study was conducted to elucidate the relationship between DNA methylation and PITX1 abnormality in AIS. The comparative analysis showed a significantly higher PITX1 methylation and lower gene expression in AIS patients as compared with healthy controls. These results further supported the results reported by Fendri et al. [12] Most importantly, our study clearly showed that the DNA methylation played an important role in the decreased PITX1 gene expression due to the relatively high promoter region methylation and this may be associated with the etiology of AIS.

To further investigate the specific effect of abnormal PITX1 methylation on the clinical and biological characteristics of AIS patients, the age, sex and Cobb angles were compared between AIS patients with positive and negative PITX1 methylation. The results revealed significant difference in age and Cobb angle of the main curves between the two groups. Bork et al. [29] reported the in-vivo hypo-methylated changes upon aging of CpGs from PITX1 promoter in mesenchymal stromal cells. The percentages of positive methylation were significantly higher in younger patients (10–13 years) than elder patients (14–16 years) in the current study, which supported the conclusion of Bork et al. [29] The rates of positive methylation in AIS patients with Cobb angle >30° were significantly higher, which showed that the DNA methylation, especially PITX methylation, might be correlated with the curve progression and curve severity. The significant correlation between methylation level of the 6 CpG sites in PITX1 promoters and Cobb angles further supported the findings. In addition, it seemed AIS boys exhibited more hyper-methylation pattern than girls even though the difference was not statistically significant (*P* = 0.308). If this phenomenon can be proven by further studies with a larger sample size, the influence of estrogen should not be ignored [30]. The role of estrogen in the regulation of PITX1 expression has been illustrated in other diseases such as breast cancer [31], however, how estrogen regulates PITX1 expression in AIS has not been reported before.

Combining the above results, we further assumed that the PITX1 promoter methylation and gene expression should be well associated. However, linear correlation analysis indicated no statistically significant correlation between PITX1 promoter methylation and gene expression (*P* = 0.842). In addition, the results of the current study showed that only 44.0% (22 of 50) patients were identified with positive methylation of PITX1 promoter. We presumed 2 possible explanations for this significant deviation. Firstly, the methylation region of PITX1 gene involved in the pyrosequencing assay was only part of the whole gene, and other fragments might also be responsible for the regulation of gene expression. Secondly, there should be other regulation pathways apart from PITX1 promoter methylation, such as the cartilage oligomeric matrix protein (COMP), protocadherin10 (PCDH10) and others [12]. Hence, further studies focusing on the methylation of other genes in AIS are indeed needed.

There were several limitations with this study. Firstly, the relatively small sample size was the largest limitation of our study. Secondly, only PITX1 promoter methylation was included in the analysis. Since it has been widely accepted that AIS resulted from combination of multiple genes, abnormalities of other regions of PITX1 gene and other potential genes was not assessed. The decision of cut-off point for positive and negative methylated subgroups based on the results of the current study was arbitrary. Nevertheless, this study put forward a novel hypothesis based on the association between genetics and epigenetics to explain the etiology and curve progression in AIS.

Recent data suggested that epigenetic responses including DNA methylation were involved not only in cellular differentiation but also in modulation of genome function in response to signals from various environments [32]. Therefore, our study not only indicated the relationship between abnormal gene methylation and occurrence of AIS but also implied the link between the Cobb angle and the aberrant methylation of PITX1 gene promoter. In addition, the results suggested that PITX1 gene methylation may be a new molecular diagnostic factor and a new biomarker for curve progression.

## Conclusions

AIS patients have significantly higher methylation level and lower PITX1 gene expression when compared to normal controls. In AIS patients, PITX1 methylation is significantly associated with the Cobb angles of main curves. This close association between genetics and epigenetics in AIS puts forward a novel hypothesis which suggests DNA methylation may play an important role in the etiology and curve progression of AIS.

## Abbreviations

AIS: Adolescent idiopathic scoliosis
PCR: Polymerase chain reaction
PITX1: Pituitary homeobox 1
